# Advanced maternal age and assisted reproductive technologies: outcomes, genomics, and real-world evidence

**DOI:** 10.3389/frph.2026.1787867

**Published:** 2026-02-26

**Authors:** C. Méndez-Vidal, M. Fernández-Sánchez, M. Domínguez-Moreno, L. García-Díaz, J. Dopazo, G. Antiñolo

**Affiliations:** 1 Institute of Biomedicine of Seville (IBIS), University Hospital Virgen del Rocío/CSIC/University of Seville, Seville, Spain; 2 Centre for Biomedical Network Research on Rare Diseases (CIBERER), Seville, Spain; 3 VIDA Recoletas, Seville, Spain; 4 Department of Surgery, University of Seville, Seville, Spain; 5 Department of Materno-Fetal Medicine, Genetics and Reproduction, Institute of Biomedicine of Seville (IBIS), University Hospital Virgen del Rocío/CSIC/University of Seville, Seville, Spain; 6 Andalusian Platform for Computational Medicine, Andalusian Public Foundation Progress and Health-FPS, Seville, Spain

**Keywords:** advanced maternal age, assisted reproductive technologies, *in vitro*fertilization, outcomes, real-world evidence, reproductive genomics

## Abstract

**Introduction:**

Advanced maternal age (AMA) and assisted reproductive technologies (ART) are becoming more common and are linked with higher obstetric and perinatal risks. Genomic tools and real-world data (RWD) are transforming risk prediction and care strategies, yet their application to the AMA + ART setting remains uneven.

**Objective:**

To synthesize recent evidence on risks associated with AMA and ART, incorporate insights from reproductive genomics and RWD, and discuss their implications for clinical care and guideline development.

**Methods:**

Narrative review of cohort studies, systematic reviews, and meta-analyses, supplemented by focused analyses of genomic approaches: expanded carrier screening, clinical exome sequencing, preimplantation genetic testing for monogenic disorders (PGT-M) and aneuploidy (PGT-A), emerging non-invasive PGT, and pharmacogenomics, as well as RWD infrastructures such as registries, EHR datasets, and trusted research environments.

**Results:**

Maternal age (≥40 years) increases miscarriage, gestational diabetes, hypertensive disorders, placental abnormalities, cesarean delivery, preterm birth, stillbirth, and NICU admissions. ART independently raises risks of preeclampsia, placenta previa, preterm birth, low birthweight, cesarean delivery, and neonatal complications, including in singleton. Combined AMA + ART may produce additive or synergistic effects. Donor oocytes reduces miscarriage risk but may elevate preeclampsia risk via immunologic mechanisms. Genomic technologies enable the identification of infertility-related variants, prevention of genetic conditions, individualized ovarian stimulation, and AI-assisted embryo selection. RWD enhances evidence by capturing diverse populations, supporting comparative and long-term analyses.

**Conclusions:**

Pregnancies from AMA + ART should be managed as high risk. Integration of genomics technologies and RWD can support predictive, personalized care and inform urgently needed consensus guidelines

## 1 Introduction

In recent decades, one of the most notable demographic changes in reproductive health has been the steady rise in maternal age at childbirth (1, 2). Social, educational, and economic factors, including higher educational attainment, career development, financial stability and evolving partnership patterns, have contributed to delayed childbearing into the late thirties and forties. This trend has coincided with advances in assisted reproductive technologies (ART), particularly *in vitro*fertilization (IVF), which have offered new opportunities for conception despite age-related fertility decline (3). However, fundamental biological constraints remain. Female fertility decreases sharply after the mid-thirties, largely due to depletion of the ovarian reserve and an increasing burden of chromosomal abnormalities in oocytes (4, 5). Advanced maternal age (AMA) is independently associated with a broad spectrum of adverse outcomes including miscarriage, hypertensive disorders of pregnancies, cesarean delivery and stillbirth (4, 6–12) ART, highly effective in overcoming infertility, introduces additional risks related both to treatment procedures and to the underlying health and reproductive characteristics of patients who require these interventions (3, 13–15).

The intersection of AMA and ART thus represents a critical challenge in contemporary reproductive medicine. Older women who conceive using ART face a double burden of risk, with overlapping mechanisms that can amplify obstetric and perinatal complications (16–19). Understanding these dynamics is essential for effective patient counseling, tailored clinical management, and the development of robust, evidence-based guidelines (7).

Genetic factors are estimated to underlie nearly half of all infertility cases, with defined genetic causes accounting for approximately 5%–10% of female and 4% of male infertility (20). Reproductive genomics offers a pathway towards personalized care. Advances in sequencing technologies, together with access to large-scale phenotype and genotype datasets, have enabled the integration of individual genomic information into reproductive medicine. However, accurate interpretation of genomic variants depends on the availability of well-phenotyped, representative reference populations.

This review synthesizes current evidence on definitions, risks, combined effects, management strategies related to AMA and ART, and explore future directions aiming to bridge scientific evidence with clinical practice.

## 2 Defining advanced maternal age

The definition of AMA has evolved over time. Historically, age 35 was considered the threshold, largely because amniocentesis was routinely recommended at this point due to a higher risk of chromosomal abnormalities (8). Although this cut-off remains widely used in clinical and research contexts, its clinical relevance has been questioned. Emerging evidence suggests that obstetrics and perinatal risks increase most sharply at age 40, prompting some experts to propose more nuanced age stratifications such as 35–39, 40–44, and ≥45 years (2, 8). The absence of a universal definition complicates comparisons across studies and meta-analyses, limiting the establishment of standardized guidelines. Moreover, the biological changes underlying age-related risk, such as oocyte aneuploidy, uterine and placental alterations, and maternal comorbidities, progress gradually rather than occurring at a fixed age (4, 10). In clinical practice, age 35 remains the conventional point at which risks begin to be emphasized, although the steepest increase is observed from 40 years onwards (2, 6). This distinction is particularly relevant for counseling women contemplating pregnancy at later ages, especially those considering the use of ART.

### 2.1 Risks associated with advanced maternal age

Miscarriage is the most prominent adverse outcomes associated with AMA. Rates rise from 15% to 20% among women aged 35–39 to about 40% at 40–44, and up to 70% in women aged 45 or older (8, 11). Oocyte aneuploidy, with chromosomal abnormalities constituting the predominant cause of early pregnancy loss, is considered the principal underlying mechanism, particularly when native oocytes are used (5). The risk of gestational diabetes also increases substantially with age. Women aged 40 or older face a three- to six-fold higher risk compared with younger counterparts (13), with prevalence approaching 20% in women over 50 (7). This reflects both age-related alterations in glucose metabolism and the higher baseline prevalence of metabolic syndrome in older women. Hypertensive disorders of pregnancy, particularly preeclampsia, represent another major concern. Prevalence increases from 3%–4% in younger mothers to 5%–10% by age 40, and up to 30% in women over 50 (9, 10). Vascular stiffness, endothelial dysfunction, and impaired placental adaptation are believed to be central mechanisms. AMA also raises the risk of placental complications, including placenta previa, placenta accreta, and placental insufficiency. These conditions increase maternal morbidity through hemorrhage and surgical complications while compromising fetal growth and oxygenation (12). Cesarean delivery rates rise sharply with maternal age. While some are elective, many procedures are prompted by medical indications such as abnormal placentation, malpresentation, and labor dystocia. Among women over 45, more than half of deliveries occur via cesarean section (6, 21). Preterm birth is more common in pregnancies affected by AMA, frequently resulting from medically indicated early delivery due to maternal or fetal complications. Stillbirth risk also rises substantially: at 39 weeks, women over 40 face a stillbirth risk comparable to that of women under 30 at 41 weeks (11). Finally, infants born to AMA mothers are roughly twice as likely to require admission to a neonatal intensive care unit (NICU), reflecting complications related to both prematurity and placental dysfunction (4, 6, 10, 12).

### 2.2 Risks associated with assisted reproductive technologies

ART have transformed infertility care, yet their benefits are tempered by clinically meaningful risks. Some complications stem from the underlying causes of infertility, while others appear attributable to ART procedures themselves (3, 14), making their relative contributions difficult to disentangle. Conditions, such as endometriosis, congenital uterine anomalies, or diminished ovarian reserve independently predispose to adverse obstetrics outcomes regardless of mode of conception. At the same time, ART-specific processes, including ovarian hyperstimulation, gametes and embryos manipulation, and embryo transfer mechanics, may exert additional influence (13). Pregnancies achieved through IVF, particularly those involving donor oocytes, carry a two- to threefold higher risk of preeclampsia (22). Proposed mechanisms include altered maternal immune tolerance to paternal or donor antigens, as well as epigenetic changes induced during ART procedures. Placental abnormalities, including placenta previa and aberrant implantation, occur more frequently in IVF pregnancies (15). It has been suggested that uterine instrumentation and the placement of embryos within the uterine cavity may alter implantation dynamics.

Importantly, elevated risks are observed even in singleton IVF pregnancies. Compared with spontaneously conceived singletons, IVF singletons have approximately a 1.5-fold increased risk of preterm birth and low birth weight, independent of multiple gestations (3, 23). Cesarean delivery rates are also markedly higher among ART pregnancies (14). While a subset of cases may reflect patient or provider preferences, many are driven by elevated rates of obstetric complications requiring surgical delivery (13). Finally, newborns conceived via ART are also more likely to require NICU admission. This vulnerability reflects a constellation of factors, including prematurity, low birth weight, and higher rates of congenital anomalies (15).

### 2.3 The combined effect of AMA and ART

When AMA and ART intersect, their associated risks appear not merely cumulative but amplified. This elevated risk profile is not limited to oocyte aging and is evident even among recipients of donor oocytes. For example, preterm birth rates rise disproportionately when both factors coexist, driven by higher rates of both spontaneous preterm labor and medically indicated early delivery (17, 18). Likewise, cesarean delivery becomes more likely, reflecting the combined burden of age-specific complications and ART-associated obstetric risk (14, 16). Maternal complications such as hypertensive disorders, gestational diabetes, and placental pathologies are also magnified in this dual-risk group (19, 22). Neonatal outcomes are similarly affected: infants born to older women conceiving through ART are more likely to be preterm, have low birth weight, and require intensive neonatal care (15, 16).

Paradoxically, some ART techniques mitigate certain risks while simultaneously introducing new ones. Use of donor oocytes, for instance, reduces miscarriage risk associated with oocyte aneuploidy but appear to increase the likelihood of preeclampsia through immune-mediated pathways (22). In support of these observations, a recent large-scale analysis of more than 33,100 good-quality single-embryo transfer cycles involving donor oocytes concluded that live birth rates declined after maternal age 40, whereas implantation failure and pregnancy loss increased after ages 39 and 43 years, respectively (24).

In summary, the elevated obstetric risk observed when AMA and ART co-occur likely reflects the convergence of three interacting mechanistic axes (i) oocyte-intrinsic aging, (ii) maternal vascular, placental, and immune alterations, and (iii) ART-related procedural and hormonal exposure. Framing the evidence within these mechanistic categories helps explain why risks may appear additive in some settings and potentially synergistic in others. A central biological feature of AMA is the progressive increase in oocyte aneuploidy, which strongly contributes to implantation failure, early pregnancy loss, and reduced live birth rates. This risk is thought to arise from age-related deterioration of meiotic competence (e.g., reduced chromosomal cohesion, spindle assembly errors) and compromised oocyte quality, including mitochondrial dysfunction, altered energy metabolism, and epigenetic drift (25–27). In autologous ART cycles, these oocyte-intrinsic mechanisms remain the dominant drivers of implantation failure and miscarriage. Importantly, these pathways primarily explain early reproductive endpoints (fertilization, embryo viability, early loss), but do not fully account for the later gestational complications that persist even when younger donor oocytes are used. Beyond oocyte quality, AMA is associated with maternal vascular and endometrial changes that can impair placentation, including endothelial dysfunction, increased arterial stiffness, and altered decidualization and spiral artery remodeling (28). These abnormalities predispose to abnormal trophoblast invasion and suboptimal uteroplacental perfusion, contributing to hypertensive disorders, fetal growth restriction, and placental complications (29). Age-related immune remodeling further compounds this risk. The concept of “inflammaging”, a shift toward low-grade systemic inflammation and immune dysregulation, may disrupt the finely balanced immune tolerance required at the maternal-fetal interface (30, 31). Advanced maternal age has been shown to alter T-cell subsets, including reductions in regulatory T-cells at the maternal–fetal interface, which may impair tolerance and contribute to adverse outcomes (32). Perturbations in regulatory T cells also influence uterine natural killer cell function and decidual vascular remodeling, processes critical for spiral artery adaptation and placental development and implicated in preeclampsia-spectrum disorders (33). Broader immune dysregulation characterized by imbalanced cytokine profiles, NK cell activation, and altered macrophage polarization has also been associated with preeclampsia (34). A particularly illustrative scenario is donor oocyte pregnancy. Although donor oocytes substantially reduce miscarriage risk by mitigating aneuploidy, they introduce greater fetal-maternal antigenic disparity (35). The increased allogeneic challenge may amplify maternal immune tolerance with downstream effects on placental development, endothelial activation, and hypertensive disease (36). Multiple cohort studies have demonstrated a 2–3-fold increase in preeclampsia risk in donor oocyte pregnancies, particularly among women of advanced age (37), and meta-analyses confirm a broader association between ART and hypertensive disorders of pregnancy (38, 39).

ART itself may add risk through both indirect and direct mechanisms. Controlled ovarian stimulation can create supraphysiologic steroid environments (particularly in fresh embryo transfers) that may adversely affect endometrial receptivity and early placentation. Differences between fresh and frozen embryo transfer (FET) cycles also appear relevant: in programmed FET cycles, the absence of a corpus luteum eliminates physiologic secretion of vasoactive/angiogenic mediators (e.g., relaxin and other luteal products), which may influence maternal vascular adaptation and contribute to hypertensive complications in susceptible individuals (40, 41). In addition, embryo culture conditions, micromanipulation, cryopreservation, and transfer technique have been proposed to influence placental gene expression and epigenetic regulation, providing biologically plausible pathways by which ART may interact with age-related vascular immune signaling vulnerability (42).

Taken together, these pathways support a coherent model in which AMA contributes to oocyte-intrinsic risk (particularly in autologous cycles) and a baseline predisposition to vascular, immune and placental maladaptation, while ART introduces additional hormonal and procedural exposures that may further perturb implantation, placentation, and maternal-fetal immune tolerance. This integrated framework helps explain the persistence of elevated adverse outcomes even in singleton pregnancies and the distinctive risk profile of donor oocyte gestations, characterized by lower miscarriage rates but higher preeclampsia risk, and underscore the need to manage AMA + ART pregnancies as inherently high risk, warranting vigilant intensified surveillance and proactive clinical management (7).

## 3 Clinical management strategies

At present, no international guidelines specifically address AMA pregnancies conceived through ART, leaving clinicians to adapt general obstetric protocols (7). Effective care begins before conception. Preconception counseling should provide couples with realistic expectations regarding success rates and risks, while also addressing alternatives such as donor gametes or adoption. Individualized risk stratification, considering maternal age, comorbidities, and fertility history, is essential in tailoring individualized care. Genetic testing offers powerful tools to refine decision-making. Preimplantation Genetic Testing (PGT) can lower the likelihood of aneuploidy in ART cycles, while Non-Invasive Prenatal Testing (NIPT) provides accurate genetic information once pregnancy is established, minimizing the need for invasive diagnostic procedures. In terms of preeclampsia prevention, daily low-dose aspirin is increasingly recommended for women at high risk, having demonstrated efficacy in reducing both incidence and severity (10). Enhanced surveillance throughout gestation is critical. This include, more frequent prenatal visits, ultrasound assessments of fetal growth and placental function, and systematic screening for diabetes and hypertension to enable early detection and timely intervention.

Delivery planning is another cornerstone of management. For women aged 40 and above, many experts recommend elective induction or cesarean delivery around 39 weeks, balancing maternal and neonatal safety while reducing the risk of stillbirth (11). To minimize the risks associated with oocyte aging, women planning to delay childbearing beyond age 35 should be informed about oocyte cryopreservation as an option. Optimal outcomes also depend on a multidisciplinary approach. Beyond obstetricians and maternal-fetal medicine specialists, the involvement of neonatologists, endocrinologists, and psychologists play critical roles. The psychological dimension should not be underestimated: older mothers undergoing ART frequently experience anxiety, guilt, or stress, which should be addressed with structured, empathetic support alongside medical management (7).

## 4 Future directions

Although considerable progress has been made in delineating the risks associated with AMA and ART, important knowledge gaps remain. Many studies lack appropriate control groups, often reporting outcomes in AMA + ART pregnancies without comparison to either age-matched women conceiving spontaneously or younger ART patients (16–19). Future research should include robust control cohorts including women of similar ages conceiving naturally and younger women undergoing ART. Large, registry-based studies capable of stratifying outcomes by both age and fertility treatment will be invaluable for disentangling the independent and combined contributions of AMA and ART. Particular attention must be paid to the growing subgroup of women aged 45 and above, who achieve pregnancy through donor oocyte IVF. Although numbers remain relatively small, their unique risk profile necessitates careful study (8, 22).

A deeper understanding of the biological mechanisms underlying adverse outcomes may eventually open the door to targeted interventions. For example, identifying placental pathways disrupted by AMA or ART could allow the development of precision therapies or tailored monitoring protocols (4, 10). Such advances could shift obstetric care from descriptive approaches to predictive and preventive strategies.

Preventive interventions, while currently limited, show promise. Low-dose aspirin is already recommended for women at high risk of preeclampsia, but further systematic evaluation of other strategies through is needed clinical trials or well-designed observational studies (10). Long-term outcomes also remain insufficiently explored. For mothers, questions persist about whether complications such as preeclampsia confer lasting cardiovascular risk when experienced at advanced age. For offspring, some evidence suggests that IVF-conceived children may exhibit slightly higher blood pressure or body mass index in childhood, but the influence of parental age on these outcomes remains uncertain (3, 13–15). Advanced paternal age, associated with infertility, miscarriage, and certain perinatal complications also merits further investigation (43).

There is also a pressing need for consensus guidelines. The absence of standardized recommendations leaves clinicians to adapt piecemeal from broader obstetric practice. International bodies such as ACOG, RCOG, and ISSM should should prioritize issuing evidence-based guidance specifically tailored to AMA + ART pregnancies (7). Finally, emerging knowledge from reproductive genomics and the use of real-world data are reshaping our understanding of human reproduction. Addressing these gaps in research, mechanistic understanding, preventive interventions, and clinical consensus, will ultimately improve outcomes for mothers and babies, ensuring that the expanding possibilities of modern reproductive medicine are matched by equally advanced standards of care.

### 4.1 Reproductive genomics

Infertility has a substantial genetic component in both women and men. In women, rare Mendelian conditions accounts for 30%–40% of infertility cases (44) while approximately 5% exhibit *FMR1* triplet repeats expansions or X-chromosome abnormalities (45). Heritable disorders such as endometriosis and polycystic ovary syndrome further contribute to female infertility. In males, chromosomal abnormalities, including Klinefelter syndrome or deletions within the azoospermia factor gene, are common causes of infertility (46). A recent genome-wide study involving 1.5 million individuals identified 22 genetic loci linked to female infertility, three to male infertility and novel variants associated with reproductive hormones, FSH, LH, estradiol and testosterone, levels, in men and women (47).

The trend toward AMA and the increasing use of ART have been linked to adverse obstetric and perinatal outcomes, with genetic factors expected to play an increasingly important role, in reduced fertility and negative reproductive outcomes (48). Reproductive genomics offers a pathway toward personalized reproductive medicine. Advances in sequencing technologies coupled with access to large-scale phenotype and genotype datasets now allow the integration of individual genomic data into clinical care. Accurate interpretation requires well-phenotyped, representative reference genomes. The Medical Genome Project, for example, uses exome sequencing of phenotyped control individuals to accelerate variant identification and facilitate the discovery of novel disease-causing genes (49, 50). Similarly, establishing a reference genome derived from healthy, fertile, phenotyped women is crucial to support personalized approaches in ART.

Clinically established genomic tools are already applied at multiple stages of ART. Preimplantation genetic testing (PGT) for aneuploidy (PGT-A), monogenic diseases (PGT-M), and structural rearrangements (PGT-SR) is widely used to assess embryo genetic status prior to transfer (26, 51), reducing the risk of miscarriage and genetic disease and supporting personalized ART protocols. Clinical exome sequencing in individuals undergoing ART and sperm donor has uncovered a substantial burden of pathogenic or likely pathogenic variants, emphasizing the growing relevance of reproductive counseling and PGT-M (52, 53). Large cohort studies further demonstrate that PGT-M, particularly when combined with comprehensive chromosome screening, is associated with improved clinical outcomes, including higher live birth rates (54). Compared to morphological evaluation alone, preimplantation genetic testing for aneuploidy (PGT-A), including non-invasive assessment using cell-free DNA from spent embryo culture medium enhances live birth and ongoing pregnancy rates per embryo transfer (55). Preconception carrier screening (56–59), is another well-established tool, although access remains limited by cost, availability to genetic counseling, infrastructures, and underrepresentation of diverse populations in genomic databases (58).

Emerging or investigational approaches include non-invasive PGT using cell-free DNA from embryo culture media or blastocoel fluid to assess chromosomal status without biopsy (60). Broader genome-wide and epigenomic analyses, including DNA methylation and transcriptomic profiling, are under investigation to improve infertility diagnosis, optimize embryo selection, and elucidate ART-associated molecular changes (61, 62). The use of polygenic risk scores is emerging to explore complex traits such as multifactorial infertility or ovarian reserve depletion (63). Although large-scale genome-wide association studies are identifying reproductive trait loci, translation into actionable clinical metrics in ART is not yet established (63). While promising, these investigational tools require further validation before routine clinical implementation.

Addressing the complexity and heterogeneity of fertility disorders, requires both genetic and non-genetic approaches. The integration of diverse data types with emerging technologies, such as artificial intelligence and machine learning, offers potential to optimize ovarian stimulation protocols, predict the likelihood of success following elective oocyte cryopreservation, and refine embryo selection to improve clinical outcomes (64). Collectively, these advances underscore the critical role of genetic testing in infertility evaluations and support its incorporation into personalized reproductive management strategies.

### 4.2 The value of real-world data in reproductive medicine

The data generated during routine clinical care and captured in sources such as electronic health records (EHRs), patient-reported outcomes, administrative claims, perinatal registries, and ART registries, known as Real-World Data (RWD), can be translated into Real-World Evidence (RWE) when analyzed using fit-for-purpose epidemiological designs and transparent reporting standards (65). RWE is increasingly recognized as a complementary evidence stream that can extend beyond the constraints of randomized clinical trials (RCTs), particularly when trials are infeasible, underpowered for rare outcomes, or poorly representative of the complex, comorbid profiles typical of AMA (66, 67).

In reproductive medicine, and especially in the AMA + ART population, the core clinical questions frequently concern: (i) Comparative effectiveness between competing strategies (e.g., fresh vs. frozen embryo transfer, elective single embryo transfer, donor oocytes, adjuvant treatments); (ii) Safety across multiple maternal and neonatal endpoints (including low-frequency but high-impact outcomes), and (iii) Longitudinal outcomes for both mother and offspring. These questions are difficult to address comprehensively in classic RCT frameworks due to sample size requirements, ethical constraints, rapid evolution of ART practice, and the need for long follow-up (67). Actually, RWD can be particularly valuable in reproductive health, where biological, social, and demographic factors intersect. For instance, AMA and ART risks may be amplified by comorbidities such as hypertension, diabetes, or obesity, conditions unevenly represented in clinical trials but readily captured at scale in healthcare databases.

RWD has been instrumental in evaluating outcomes that are either uncommon or require very large denominators. For instance, large registry-linked analyses have compared perinatal outcomes after frozen vs. fresh embryo transfer, including sibling-comparison designs that partially control for stable maternal factors and strengthen causal interpretation (68). These real-world comparative analyses are directly relevant to AMA, where clinical decisions are frequently individualized and where balancing maternal hypertensive risk, placental complications, prematurity, and neonatal growth patterns may differ across treatment strategies.

An example of generation of evidence from RWD, that has already yielded novel insights in women's health, is the Andalusian Collaborative Environment for Advancing Predictive Care (69). This environment allows researchers carrying out ethical and secure research with clinical data from the Andalusian Health Population Database (BPS) within a trusted research environment, to generate evidence under strict data protection and GDPR compliance, ensuring scalability and reproducibility. Currently, BPS integrates detailed longitudinal clinical data from more than 15 million residents of Andalusia, which provides an idea of the sample sizes available for studies. In this environment. large-scale population-based studies have shown how menopause significantly modifies the trajectory of chronic diseases, with postmenopausal women exhibiting disproportionate increases in hypercholesterolemia, osteoporosis, hypertension, and anxiety disorders compared to men of similar age, using a cohort of more than 550,000 patients (70). These findings highlight how hormonal transitions can be studied in real-world populations, providing actionable knowledge for prevention and management strategies. Similar approaches can be applied to study the combined effects of AMA and ART, generating robust, population-level evidence that complements genomic studies and clinical trials.

Similarly, predictive modeling using RWD has advanced early detection strategies. Our group recently demonstrated that machine learning applied to population health databases can identify women at elevated risk of ovarian cancer well before diagnosis, paving the way for more personalized surveillance and preventive interventions (71). Such approaches are directly relevant to AMA and ART, where early recognition of maternal risks could inform counseling, treatment choices, and follow-up intensity. In the ART setting, RWD can be leveraged to compare outcomes of autologous vs. donor oocyte cycles, fresh vs. frozen embryo transfers, or different stimulation protocols in real-world practice. Importantly, large-scale databases can also track maternal and offspring outcomes beyond the perinatal period, addressing current gaps regarding long-term cardiovascular or metabolic risks after ART pregnancies.

### 4.3 Toward evidence-based guidelines

There is an urgent need for dedicated guidelines for pregnancies in AMA women undergoing ART. RWD can play a central role in closing this gap by:

- Providing comparative effectiveness evidence between ART modalities in older women
- Stratifying risk by maternal age, comorbidities, and treatment characteristics,
- Capturing long-term maternal and offspring outcomes
- Enabling rapid, iterative evidence generation through secure, federated data infrastructures.

By integrating genomic data with large-scale RWD, reproductive medicine can move from descriptive associations to predictive, preventive, and personalized care. This synergy has the potential to generate actionable knowledge, guiding both clinical practice and health policy in an area where evidence is currently fragmented.

## 5 Conclusions

Advanced maternal age and assisted reproductive technologies represent two of the most defining trends in contemporary reproductive medicine. Each independently heightens the risks of obstetric and perinatal complications, but their combined effects are often compounded. Women aged 35 and older, especially those over 40, who conceive through ART face an increased likelihood of miscarriage, preeclampsia, gestational diabetes, placental disorders, cesarean delivery, preterm birth, and neonatal morbidity (1–16). The use of donor oocytes or previously frozen autologous oocytes cryopreserved before age 35, may partially mitigate age-related declines in fertility and reduce miscarriage risk; however, they do not eliminate maternal health risks and may, in some cases, susch as preemclamsia, introduce additional challenges (22). For clinicians, this underscores the importance of comprehensive preconception counseling, individualized and vigilant monitoring throughout pregnancy, and carefully planned delivery strategies. For researchers, the priority is to elucidate the biological mechanisms underlying these risks and to develop evidence-based multidisciplinary approaches to safeguard both maternal and neonatal outcomes (7).
